# Touch Influences Visual Perception with a Tight Orientation-Tuning

**DOI:** 10.1371/journal.pone.0079558

**Published:** 2013-11-14

**Authors:** Onno van der Groen, Erik van der Burg, Claudia Lunghi, David Alais

**Affiliations:** 1 Department of Cognitive Psychology, Vrije Universiteit Amsterdam, Amsterdam, The Netherlands; 2 School of Psychology, University of Sydney, Sydney, Australia; 3 Department of Neuroscience, Psychology, Pharmacology, and Child Health, University of Florence, Florence, Italy; 4 Institute of Neuroscience, National Research Council (CNR), Pisa, Italy; Bielefeld University, Germany

## Abstract

Stimuli from different sensory modalities are thought to be processed initially in distinct unisensory brain areas prior to convergence in multisensory areas. However, signals in one modality can influence the processing of signals from other modalities and recent studies suggest this cross-modal influence may occur early on, even in ‘unisensory’ areas. Some recent psychophysical studies have shown specific cross-modal effects between touch and vision during binocular rivalry, but these cannot completely rule out a response bias. To test for genuine cross-modal integration of haptic and visual signals, we investigated whether congruent haptic input could influence visual contrast sensitivity compared to incongruent haptic input in three psychophysical experiments using a two-interval, two-alternative forced-choice method to eliminate response bias. The initial experiment demonstrated that contrast thresholds for a visual grating were lower when exploring a haptic grating that shared the same orientation compared to an orthogonal orientation. Two subsequent experiments mapped the orientation and spatial frequency tunings for the congruent haptic facilitation of vision, finding a clear orientation tuning effect but not a spatial frequency tuning. In addition to an increased contrast sensitivity for iso-oriented visual-haptic gratings, we found a significant loss of sensitivity for orthogonally oriented visual-haptic gratings. We conclude that the tactile influence on vision is a result of a tactile input to orientation-tuned visual areas.

## Introduction

To gain a more unified and accurate perception of the world, information from different sensory modalities are frequently integrated [1]–[3]. The classical view of multisensory processing is that unimodal signals are first encoded separately in unisensory cortices and that any integration across sensory modalities occurs subsequently in higher-level association areas. However, research evidence has accumulated over the last decade, which indicates that sensory processing may be more multisensory than initially believed, with previously assumed ‘unisensory’ areas displaying multisensory characteristics [2], [4]–[8]. Support for this view comes from recent behavioural studies demonstrating that information in one modality can affect perception of signals presented in other sensory modalities. For example, a sound can enhance performance on a purely visual task, even when the sound is spatially non-informative [9]–[11], and a single flash can appear as a double flash if accompanied by a double ‘click’ sound [12].

Interactions have not only been found between sound and vision, but also between touch and vision. For instance, Van der Burg, Olivers, Bronkhorst &Theeuwes [13] and Violentyev, Shimojo & Shams [14] demonstrated an interaction between vision and touch in the temporal domain. In the Van der Burg *et al.* [13] study, participants were required to search for a horizontal or vertical line among a cluster of distractor line segments of different orientations. All lines in the display underwent a periodic colour change however each changed at a different point in time. When the colour change of the target line co-occurred with a brief tactile stimulus, search times and search slopes for the target were significantly reduced, even though the tactile stimulus was uninformative about the target's location, orientation and colour. Violentyev *et al.* [14]showed a touch-induced flash illusion, similar to the sound-induced flash illusion in the audiovisual domain [12]. Participants reported seeing two flashes when two task-irrelevant tactile stimuli were presented concurrently with a single flash. Furthermore, the sensitivity *(d′)* for detecting the visual stimulus increased, suggesting a genuine sensory interaction between touch and vision. Neuroimaging studies also show interactions between tactile and visual processing. For example, studies in blind people demonstrate activation in early visual cortices during Braille reading [15]–[20]. Activation of early visual cortices has also been demonstrated in sighted subjects after short-term visual deprivation [21], consistent with the idea that connections between visual and tactile processing areas become more effective in the absence of visual input [22]. Another study examined tactile discrimination thresholds and showed that an accompanying visual stimulus enhanced tactile discrimination and suggested an additive combination of visual and tactile signals [23]. A decrease of tactile discrimination thresholds with non-informative vision of the finger has also been demonstrated [24], providing further evidence for visual tactile interactions in early sensory cortices.

More recently, studies by Lunghi, Binda & Morrone [25] and Lunghi and Alais [26] showed an interaction between vision and touch in binocular rivalry. During binocular rivalry, two different but equally salient stimuli are presented to each retina and subjects experience a bistable visual percept. At any given moment, only one of the images is consciously perceived and the other image is suppressed. This suppression is believed to include early stages of visual processing, including V1 where left- and right-eye signals are first combined [27], [28]. In the Lunghi *et al.* [25] study, two visual gratings with orthogonal orientations were used (one presented to each eye) to produce binocular rivalry. Participants were asked to continuously indicate which of the two gratings was being perceived. Subjects were intermittently instructed to explore a haptic grating for several seconds, which had the same spatial frequency and orientation as one of the visual stimuli. The data showed that active exploration of the haptic stimulus extended the dominance duration of the matching visual stimulus if it was currently dominant, or reduced dominance duration when the other grating was dominant so that perception quickly changed to match the haptic stimulus. In a subsequent paper, Lunghi and Alais [26] showed that this visual-haptic interaction in rivalry exhibits a tight orientation tuning, with the effect declining rapidly as the orientation difference between the haptic and visual gratings increases. Several other studies also have shown evidence of visual-tactile interactions [14], [29], [30].

The orientation dependence in the visual-haptic interaction in Lunghi *et al.*'s study [25] is striking, as is the fact that it was found to be spatial-frequency tuned (such that a haptic grating differing by one octave from the visual gratings did not produce the effect). Lunghi *et al.*'s [25] results imply that the interaction likely occurs at an early stage of cortical processing where visual neurons are tuned for basic stimulus attributes such as orientation and spatial frequency. Although the reported orientation and spatial frequency tunings reported in the study by Lunghi and colleagues [25] are suggestive of an early sensory interaction, the binocular rivalry paradigm is inherently subjective as observers must report their own perceptual states and is therefore open to response bias.

In the current study, we test for a genuine visual-haptic integration by investigating whether touch can influence visual contrast sensitivity using a two-interval, two-alternative forced-choice design to eliminate response bias. In addition, the orientation (Experiment 2) and spatial frequency (Experiment 3) will be systematically manipulated to examine whether visual-haptic interactions occur in visual processing areas that encode those stimulus attributes [31].

## Experiment 1

In this experiment we tested the hypothesis that task-irrelevant haptic stimulation can influence visual contrast sensitivity in a two-interval detection task. Participants were asked to indicate in which of two intervals a visual target stimulus was presented, regardless of its orientation. The target was an oriented grating (45° clockwise, or 45° counter clockwise) and the contrast of the target stimulus was adjusted after each trial using an adaptive staircase procedure (QUEST) [32] in order to find the contrast threshold for detecting it. The visual and haptic stimuli were spatially collocated and matched in spatial frequency, although the haptic orientation could either be *congruent* (same orientation as the visual stimulus) or *incongruent* (orthogonal to the visual stimulus). We expected to find a lower visual detection threshold in the congruent condition compared to the incongruent condition.

### Materials and Methods

#### Ethics statement

This research was approved by the University of Sydney Human Research Ethics Committee. Written consent was obtained from each participant prior to the experiments. The experiments were approved by the local ethics committee of the University of Sydney.

#### Participants

Thirteen subjects participated in this experiment (7 males, aged between 23–46 years, M = 26.5). Nine subjects were not aware of the hypothesis being tested. One of the subjects was left handed, however all subjects conducted the haptic exploration with the index finger of their dominant hand. All subjects had normal or corrected-to-normal vision.

#### Stimuli and apparatus

**Figure 1:**
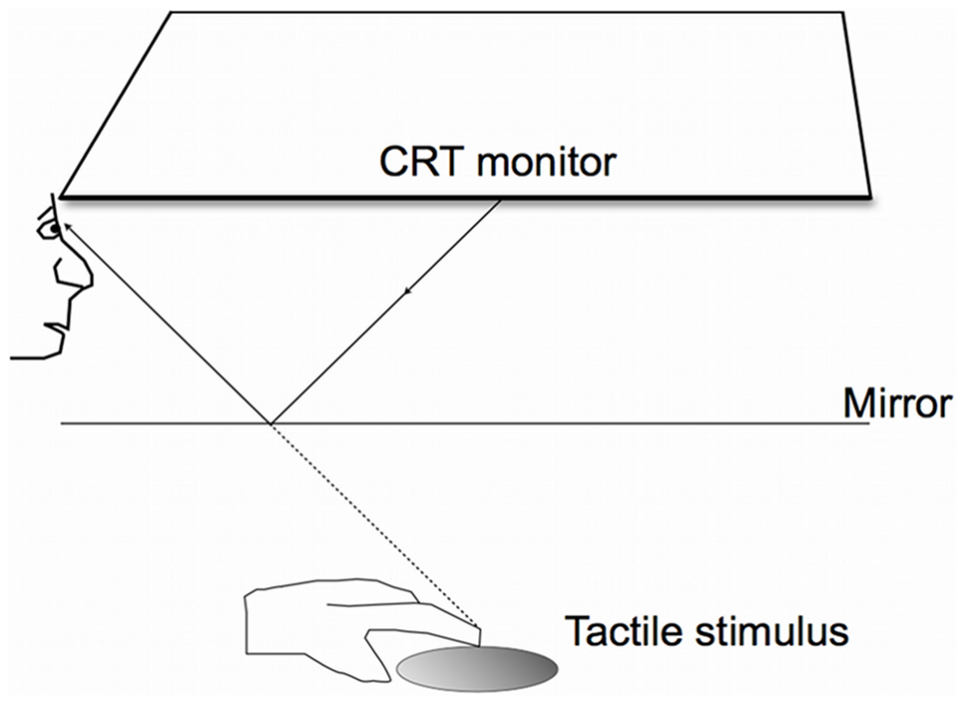
Experimental setup. Subjects explored the haptic grating with the index finger of their dominant hand while looking into a mirror which imaged a visual grating in a collocated position and with the same spatial frequency. Subjects could not see their hand through the mirror.

Visual stimuli were generated using Matlab version 7.10.0 (Natick, MA, 2010a) and the Psychophysics toolbox [33]–[35]. Stimuli were controlled by a Mac Pro computer desktop running Mac OSX (Apple Computers, Cupertino, CA). Stimuli were presented on a Mitsubishi Diamond Pro 2070SB colour monitor with a calibrated, linearized output at a resolution of 800×600 pixels, with a refresh rate of 100 Hz. The monitor was mounted on top of a wooden box (53×52×44 cm) with the screen facing downwards (see Figure 1). The orientation of the haptic stimuli was controlled by an Arduino Uno stepper motor and a microcontroller board (Arduino) in Matlab using the Matlab support package for Arduino (Mathworks, 2009). A mirror (41.5×40 cm) was placed halfway between the monitor and the haptic stimulus in the horizontal plane, so that the image reflected from the monitor appeared to be coming from the same spatial location as the haptic stimulus. The visual and haptic stimuli were, thus, spatially collocated.

**Figure 2:**
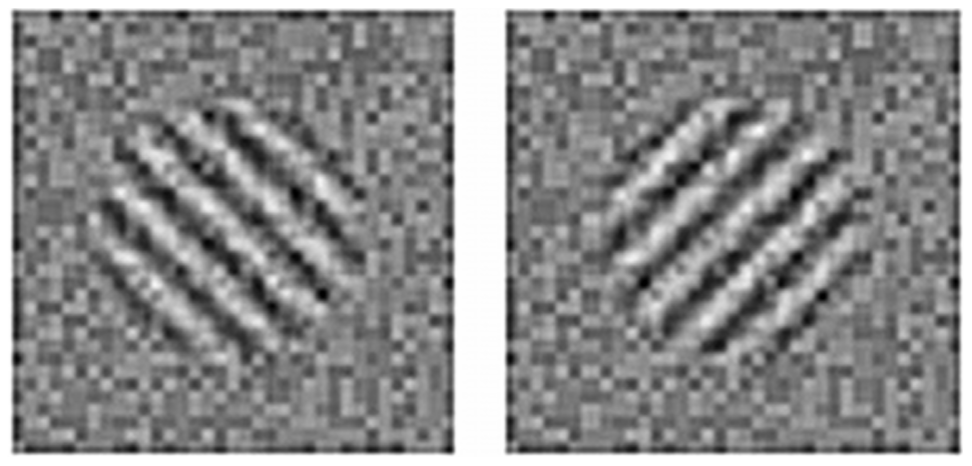
Visual stimuli. The visual target stimulus was a low contrast grating added to a background of random visual noise of 20% contrast. The grating could be presented counterclockwise or clockwise by 45° relative to vertical. In this figure, the visual target is shown at suprathreshold contrast, although in the experiment the contrast was much lower and was adjusted over trials to find the contrast detection threshold. In the non-target interval, only the noise was present, and the order of target and null intervals was randomized over trials.

The visual target stimulus (see Figure 2) was a Gabor patch (visual angle 2.90°, spatial frequency 2.66 cycles per cm, mean luminance 65 cd/m^2^) in a soft circular aperture with an orientation of either 45° clockwise or 45° counter clockwise. The Gabor patch was embedded in static-random white noise (noise contrast 20%). The Gabor patch was presented with a temporally smooth on- and offset. This was produced by multiplying the stimulus by a Gaussian temporal profile with a standard deviation of 23.33 ms. Presented in a randomized order, one interval contained the Gabor patch plus noise, and the other interval contained only the noise. Both stimuli were presented on a black background (2.6 cd/m^2^) in the centre of the screen.

The haptic stimulus was a round disc (diameter 3 cm) with sinusoidal corrugations whose spatial frequency matched that of the visual stimulus (2.66 cycles per cm). The haptic stimulus was created with a 3D-printer. The haptic stimulus was mounted transversally on the Arduino motor shaft so that the haptic grating could change orientation and the motor was placed at the bottom of the box at the position overlapping with the apparent location of the visual stimulus. Subjects could not see the haptic stimulus nor their hand touching it.

#### Procedure

**Figure 3:**
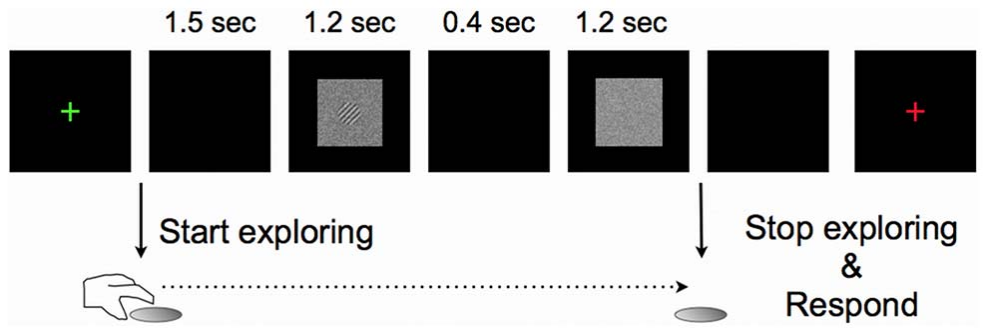
Experimental procedure. The visual target stimulus, 45° clockwise or counterclockwise (here clockwise) could be presented in the first or second interval. In this figure, the stimulus is shown in the first interval at suprathreshold contrast. After the green fixation cross disappeared, participants started to explore the haptic grating with the index finger of their dominant hand. Two intervals were presented on the screen, separated by a blank screen for 40 ms. After the second interval disappeared, subjects had to indicate in which interval the visual grating was presented. The grating's orientation alternated randomly between +/−45°. After the response a red fixation-cross appeared, indicating that the participant was not allowed to touch the grating. The motor made two random turns before the next trial started.

The experiment took place in a dark and quiet room. Subjects were first familiarised with the task by viewing the visual stimulus in full contrast in 5 practice trials. Each trial started with a green fixation cross (visual angle 0.6°). After a key-press, the fixation cross disappeared and the subject started to explore the haptic grating by making circular motions with their index finger. After 1.5 seconds, two intervals were presented on the screen for 1.2 s each, separated by a blank interval of 40 ms. Each interval started with 25 ms of random white noise frames and ended with 25 ms of random white noise frames. One of the intervals contained the visual grating embedded in noise and the other contained white random noise (Fig. 3). The same random noise was used in each interval for a given trial. The visual grating was presented at random in the first or second interval. After the second interval disappeared, subjects stopped exploring the haptic stimulus and were asked to make a forced-choice judgment to indicate the interval containing the visual grating. After their response, a red fixation-cross appeared on the screen, indicating they were not allowed to touch the grating. The haptic grating made two random turns before reaching the final position for the next trial to ensure the noise from the motor did not provide a cue for what the next orientation would be. Congruent and incongruent trials were presented randomly within a session.

The contrast of the visual stimulus was varied logarithmically over trials using the adaptive QUEST [32] procedure driven by performance on the 2AFC task. Two QUEST staircases were randomly interleaved, one for the congruent condition and one for the incongruent condition, with 40 trials per QUEST. All subjects performed three sessions, making a total of 120 trials per condition.

#### Analyses

Data from each observer's three sessions were pooled and contrast thresholds corresponding to 75% correct detection were determined for each condition (congruent and incongruent). To determine the 95% confidence intervals (CI) for each threshold, a bootstrap procedure was used: the data were resampled 1000 times and a cumulative Gaussian distribution was fit to each resampled data set. From the resulting distribution of 1000 resampled thresholds, the points bounding the central 95% of means defined the upper and lower CIs. The contrast thresholds were then compared using a paired t-test. The average slopes of the psychometric functions in each condition were also compared with a paired t-test.

### Results and Discussion

**Figure 4:**
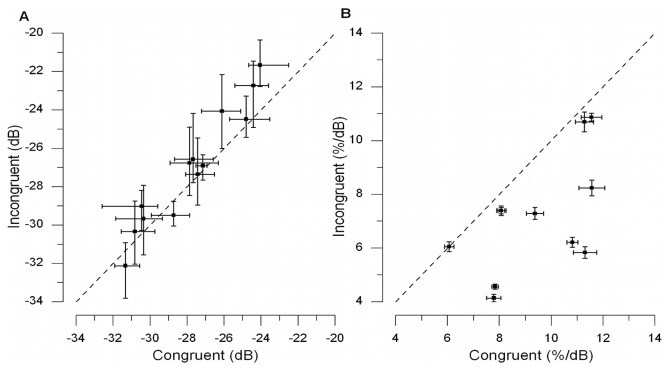
Results of experiment 1. A) Scatterplot of data from individual subjects showing the relationship between the thresholds for the congruent and incongruent condition. The dotted line depicts a linear relationship between the congruent and incongruent condition. Most subjects show a lower visual threshold for the congruent condition than for the incongruent condition. Error bars show the 95% CI for each subject's bootstrapped data. The visual contrast thresholds where converted from a linear contrast scale to a decibel (dB) scale using the following equation: *Contrast* (*dB*) = 20*log_10_(*Linear Contrast*). B) Scatterplot of the relationship between the slopes for the congruent and incongruent condition. The dotted line depicts a linear relationship between the congruent and incongruent condition. Most subjects have a steeper psychometric curve for the congruent condition than for the incongruent condition. Error bars show the 95%-CI for each subject of the bootstrapped data.

Two tailed t-tests were used to examine whether the observed congruency effects were different for the two sets of orientations used in the study (i.e., left-tilted vs. right-tilted). This analysis yielded no reliable difference in terms of slope and threshold and so the data sets were merged for further analysis. The group mean data (Fig. 4) show that the visual contrast threshold for the congruent condition (mean = −27.9 dB, SD = 2.44) was significantly lower than the threshold for the incongruent condition (mean = −26.9 dB, SD = 3.08), *t*(12) = −3.43, *p = .005*, (paired t-test, Fig. 4-A). These results support our hypothesis; when the haptic grating shares the same orientation as the visual grating, haptic stimulation can lower visual contrast sensitivity compared to when the orientations differ from each other. The slope of the psychometric function is a measure of sensitivity of the observer, with a steeper function implying a higher sensitivity. Our data show a higher visual sensitivity when touching a haptic stimulus congruent in orientation with the visual stimulus (i.e., higher slope mean = 0.0968, SD = 0.019) compared to the visual sensitivity measured during haptic exploration of a haptic stimulus incongruent in orientation with the visual stimulus (i.e., lower slope: mean = 0.0698, SD = 0.0203), *t*(12) = 4.83, *p*<.001) (Fig. 4-B). Furthermore, the results obtained cannot be explained by a response bias, because participants were required to touch the same grating during both intervals, and asked to detect the visual target regardless of its orientation.

## Experiment 2

In Experiment 1, we established that congruent, haptic stimulation lowered visual contrast detection thresholds and improved visual sensitivity as compared to incongruent haptic stimulation. In Experiment 2, we tested whether the haptic influence of visual sensitivity when exploring a congruent grating compared to exploring an incongruent grating that we observed in Experiment 1 was tuned for fine orientation. As in the previous experiment, participants were asked to indicate in which of two intervals a visual target stimulus was presented while simultaneously exploring a haptic grating that was collocated with the visual stimulus and was matched in spatial frequency. The contrast of the visual target stimulus was fixed for observers at the level of their contrast threshold. For those observers who completed Experiment 1, the threshold from that experiment was used. Several additional observers completed a similar contrast threshold experiment before beginning Experiment 2. In Experiment 2, the orientation of the haptic grating varied from trial to trial so that the orientation tuning of the visual-haptic interaction could be mapped out. If our data support the hypothesis that the visual-haptic interaction occurs in cortical areas that contain orientation-selective neurons, then detection of the visual stimulus will depend on the orientation of the haptic explored stimulus. Performance should be maximal when there is no difference in orientation between the visual and haptic gratings, and as the relative orientation difference increases in a positive or negative direction performance should fall off, consistent with the tight orientation tuning that is only observed in early visual areas.

### Materials and Methods

#### Participants

Thirteen subjects participated in this experiment (nine males, age range of 23–37 year, mean = 24.5, 2 left-handed). Eleven subjects were not aware of the hypothesis being tested. All subjects conducted the haptic exploration task with their dominant hand, and all had normal or corrected-to-normal vision.

#### Procedure

The experimental procedure was the same as in Experiment 1 with a few changes in stimuli and apparatus. The contrast of the visual stimulus was fixed in this experiment: for each individual, the contrast was determined by averaging the 75% contrast threshold for the congruent and incongruent conditions in Experiment 1. The haptic stimulus was presented at a range of orientations, 6 clockwise from vertical (15°, 35°, 40°, 45°, 50°, 70°) for the right-handed participants and 6 counter-clockwise orientations from vertical (15°, 35°, 40°, 45°, 50°, 70°) for the left-handed participants. The visual orientation was either 45° clockwise or 45° counter-clockwise, 6 haptic orientations were tested, 12 times per block. The order of the haptic orientations presented was randomized, as was the alternation of the visual orientation between clockwise and counterclockwise. Each subject conducted 1 block of 144 trials.

### Results and Discussion

**Figure 5:**
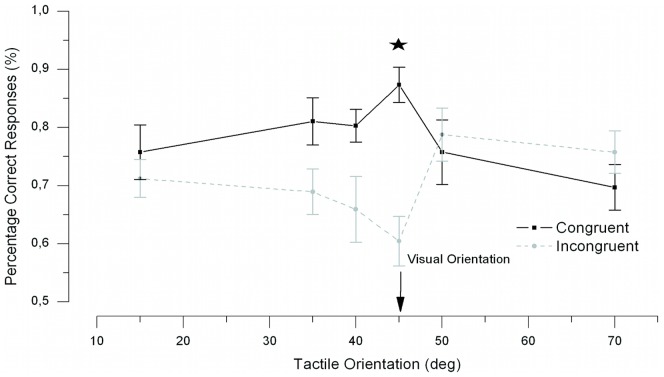
Results of Experiment 2. The orientation tuning curve. The data show a significant main effect of congruency and a significant interaction between congruency and tactile orientations. The star indicates a significant difference between congruent and incongruent conditions. The curves only differ significantly when the haptic orientation is 45°, the point at which it was perfectly aligned with the visual grating (also at 45°). Error bars showing standard error of the mean.

Two participants were excluded from the analyses because their preliminary estimate of contrast produced performance that differed greatly from 75%. One of them performed near chance level (averaging 54% correct) and the second showed a ceiling effect (averaging 93% correct). This left a total number of 11 participants in the analyses. The results of Experiment 2 are presented in Figure 5. Mean accuracy was subjected to an ANOVA with congruency and orientation as within subject variables. The main effect of orientation was not significant (*F*<1). The main effect of congruency was significant, *F* (1, 10) = 7.43, *p* = .021, indicating that the overall performance was better in the congruent condition than in the incongruent condition. Importantly, the ANOVA yielded a significant interaction between congruency and orientation, *F*(5, 50) = 5.92, *p*<.001, demonstrating that the congruency effect depends on the orientation of the haptic stimulus. The congruency effect was further examined for each haptic orientation by pairwise two-tailed t-tests. The t-tests (Bonferroni corrected for multiple comparisons), yielded a significant congruency effect when the haptic orientation was 45°, *t*(5) = −6.217, *p*<.001, but not for any other haptic orientations (all *ps*>.079). The effect of orientation was further examined by separating the data into congruent and incongruent trials and conducting separate ANOVAs on each to examine whether 45° differed significantly from the other orientations. The ANOVAs yielded significant main effects of orientation in both the congruent and the incongruent conditions, F(5,50) = 3.482, p = .009, and F(5,50) = 3.923, p = .004, respectively. To verify that this effect is indeed driven by the tactile and visual orientation of 45°, we tested the effect of tactile orientation without the tactile orientation of 45°. This analyses showed no significant effect of orientation for both the congruent and incongruent orientations (all *ps>.092*. This confirms the presence of a significant peak at 45° where the haptic-visual gratings are iso-oriented (i.e., congruent), and a significant trough at 45° for orthogonal haptic-visual gratings (i.e., incongruent).

## Experiment 3

The results of Experiment 2 showed that haptic influence on visual contrast sensitivity depends on the orientation of the haptic stimulus and is only significant when the visual and haptic gratings share the same orientation. In this experiment, we tested whether the haptic enhancement effect is spatial frequency tuned. Spatial frequency tuning is a key property of neurons in early visual cortices [36]. The experimental procedure was as used in the previous experiments, a two-interval design with visual contrast fixed at threshold level but with the haptic grating varying in spatial frequency above and below the frequency of the visual stimulus. If the data support the hypothesis, then visual detection performance will depend on the spatial frequency of the haptic grating.

### Materials and Methods

#### Participants

Thirteen subjects participated in this experiment (nine males, age range of 23–37 years, mean = 24.5, 2 left-handed). Eleven subjects were not aware of the hypothesis being tested.

#### Procedure

The experimental procedure was identical as in Experiment 2, however stimuli and methods differed slightly. The haptic stimuli were five sinusoidal gratings with a diameter of 3 cm. The spatial frequencies used were 1, 1.33, 2.66, 4 and 8.33 cycles per cm. Only one of the haptic gratings matched in spatial frequency with the visual stimulus (i.e., 2.66 cyc/cm). The haptic stimuli were attached to a large disk and were equally spaced around it, and mounted on the steppermotor that rotated the disk to locate a given haptic grating under the observer's dominant hand, allowing easy and automated switching among haptic stimuli between trials.

The haptic stimulus was presented at 45° clockwise for the right-handed participants and at 45° counter-clockwise for the left-handed participants. The visual orientation was either 45° clockwise or 45° counter-clockwise, 5 haptic spatial frequencies were tested, 14 times per spatial frequency for both visual grating orientations. Each participant conducted one session of 140 trials. The order of the haptic spatial frequency presented was randomized, as was the alternation of the visual orientation between congruent and incongruent.

### Results and Discussion

**Figure 6:**
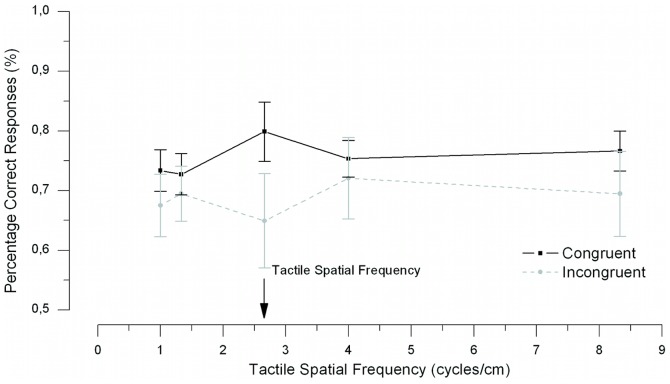
Results of Experiment 3. The spatial frequency tuning curve. The data show a significant main effect of congruency. Error bars showing standard error of the mean.

Two participants were excluded from the analyses because their performance did not exceed chance (53% and 57% correct, on average). This left 11 participants in the analyses. The results of Experiment 3 are presented in Figure 6. Mean accuracy was subjected to an ANOVA with congruency and spatial frequency as within subject variables. The ANOVA yielded a significant main effect of congruency *F*(1, 10) = 6.69, *p* = .027. This congruency effect was not dependent on the spatial frequency, as the two-way interaction was far from significant (*F*<1).

## General Discussion

Recent research has demonstrated that stimulation in one modality can influence the activation of unisensory areas belonging to another modality. Intersensory integration between touch and vision has been demonstrated in several studies [13], [14], [23], [25], [26]. Our results are in line with these studies reporting an interaction between vision and touch. In our experiments, the possibility of a response bias was eliminated as the same haptic grating was presented during both intervals, and participants were asked to detect the visual target regardless of its orientation. The results of Experiment 1 established that visual contrast sensitivity in a grating detection task improved when participants simultaneously explored a haptic grating with the same orientation as the visual stimulus, relative to their visual sensitivity when exploring an orthogonally orientated haptic grating. We showed in Experiment 2 that this effect depends on the orientation of the haptic stimulus and is maximal when the visual and haptic gratings share the same orientation. In Experiment 3 we replicated the congruency effect from Experiments 1 and 2 and showed that this effect is not tuned for spatial frequency.

Could this haptic facilitatory congruency effect of haptic input on vision be due to attention? It is known from several studies that visual contrast sensitivity can be increased by attention [37]–[41]. It may be argued, for example, that participants in our task attended to the haptic grating (perhaps automatically due to its novelty, or intentionally as they performed their scanning movements), however this seems unlikely as we show that the congruency effect critically depends on the orientation of the haptic grating. A simple attention-based account does not predict a narrow orientation dependency. Indeed, narrow orientation tunings are a typical property of neurons in the early visual cortex [42] and the results of Experiment 2 therefore suggest an early interaction between vision and touch, possibly in V1.This is in line with the narrow orientation tuning for visual-haptic interaction reported by Lunghi and Alais [26], and also with studies showing that early visual cortex is activated by tactile stimuli in blind individuals [20], [43]. Interestingly, early visual cortex (bilateral occipital region that including the calcarine scissure) has been shown to be activated by tactile stimulation in normally sighted observers after 5 days of blindfolding [22], activation that was accompanied by increased performance in haptic discrimination of Braille letters. Shorter-term visual deprivation (up to 110 minutes), has instead been shown to be ineffective in facilitating haptic discrimination performance [44].

Top-down effects from imagery on haptic shape perception have been previously documented [45]–[49] but these mostly engage imagery of higher-level visual features as object size, shape, mental rotation (reviewed in Kosslyn *et al.* [48]), and in fact visual imagery is often associated with visual short-term memory [48]. However, it has been demonstrated that visual imagery can elicit neural activity in orientation-selective visual areas ([50]) leading to both perceptual and neural adaptation, and that imagery of visual orientation is involved in tactile perception [51]–[53]. Even though imagery of haptic orientation could, in principle, play a role in mediating the haptically-driven facilitation of visual contrast thresholds that we observed, the tight orientation tuning of the effect here argues against a major role for imagery. Our result suggests that the orientation selectivity of the visuo-haptic interaction is unlikely to be explained in terms of imagery or categorical top-down decision. Another result pointing to a low-level explanation is the loss of visual sensitivity for orthogonally oriented visual-haptic gratings (Fig. 5). This loss of visual sensitivity is consistent with cross-orientation inhibition, a cross-modal version of an effect documented in V1 neurons in which the firing-rate of an otherwise optimally driven, orientation-selective V1 neuron is reduced by a superposed orthogonal grating whose orientation fails to drive the neuron when presented alone [54], [55]. Cross-orientation inhibition is thought to occur intracortically in V1, and so would be consistent with the loss of visual sensitivity reported for orthogonally oriented visual-haptic gratings. A final reason to support an early account of our orientation findings is a result by Lunghi and Alais [26] in which visual-haptic gratings differing in orientation by as much as 15° were frequently judged to be identical. This suggests poor conscious awareness of small orientation differences between touch and vision. There are reports that show a role for higher-level decision areas on perceptual performance in perceptual learning tasks [56]–[58] after prolonged training and with a long time interval between the training sessions. However, it is unlikely that our findings are the result of perceptual learning as it has been suggested in visual perceptual learning tasks, that a consolidation period of 8 to10 hours is necessary between training sessions for learning to occur [59], [60]. Participants in our study did not have a consolidation period within the different experiments. Moreover, the congruency effect at a tactile orientation of 45° in Experiment 3 is not larger than the congruency effect at the same tactile orientation in Experiment 2. If we were to measure a practice effect, we would expect a larger effect in Experiment 3 than in Experiment 2 as the participants had more practice in Experiment 3. A high-level, top-down account would require awareness of the orientation differences to provide a tenable alternative.

One of the most intriguing aspects of this study is the tight orientation tuning of the interaction between vision and touch. Even though high-level visual areas such as the parahippocampal place area [61] and face-sensitive areas of the fusiform gyrus [62] have been demonstrated to be selective to low-level visual characteristics (e.g., spatial frequency selectivity), a narrow orientation tuning is a typical property of early visual neurons [42], [63] that is not shown by higher-level visual areas. The broadening of receptive field properties along successive stages of visual processing means that high-level visual areas (e.g., PPA [64]) show a preference for cardinal orientations (i.e., an “oblique effect”) but not a specific fine orientation tuning. Although we cannot directly derive conclusions about the neural locus of the interaction between vision and touch from our experiments, our finding of a tight orientation-tuned interaction between vision and touch may suggest that tactile signals can reach early visual areas (possibly V1), producing the observed detection facilitation for visual gratings sharing the same orientation.

In addition to orientation tuning, a further property of neurons in early visual cortex is spatial-frequency tuning [31]. Experiment 3 showed a facilitation of visual detection for congruently aligned haptic gratings compared to incongruently aligned haptic gratings, however it was not spatial-frequency tuned. The significant congruency effect in Experiment 3 confirms that visual and haptic stimuli sharing the same orientation is critical for eliciting the haptic-visual interaction. The fact that we find a tuned effect for orientation but not for spatial frequency could mean two things. First, it could indicate that orientation is more critical for eliciting the relative haptic-visual contrast enhancement. The reason for this may be that visual spatial frequency is dependent on viewing distance whereas spatial texture on haptic surfaces is not. This means the relationship between visual and haptic spatial frequency is variable and not a robust cue to visual-haptic integration. Second, it is possible that the interaction between vision and touch does not occur in early visual cortices where neurons are tuned to both orientation and spatial frequency but occurs at a later stage of visual processing. Area V6, for example, has been shown to be activated by a tactile orientation task [65]. Imaging or transcranial magnetic stimulation (TMS) studies are required to make clear which level the interaction between vision and touch occurs at.

An unexpected but interesting finding was the trough in the tuning curves in Experiments 2 and 3 when touch and vision were incongruently (i.e., orthogonally) oriented (see Figs. 5 and 6). A possible explanation for this dip in performance in the incongruent condition is cross-orientation inhibition in the visual cortex [66]–[68]. Cross-orientation inhibition has been shown in animals and, more recently, in human visual cortex [69]. It occurs when two visual gratings are presented, one at a neuron's preferred-orientation and one at the orthogonal orientation. Relative to the response when the preferred grating is presented alone, a smaller response occurs when an orthogonal orientation is added [70]. This response decrease occurs even though the orthogonal grating is well beyond the neuron's orientation bandwidth, and is presumed to be due to an inhibitory signal between orthogonal orientations. In our study, the incongruent condition involved orthogonal haptic and visual gratings (e.g., touch 45° and vision 135°). As the visual signal was presented at threshold and was therefore weak, any haptic input to early visual cortex would be strong in relative terms and, being orthogonal, might be sufficient to elicit cross-orientation inhibition. Such an account would explain the dip in the tuning curves in Figures 5 and 6 and leads to a testable prediction that cross-orientation inhibition can be elicited cross-modally. This hypothesis could be ideally tested in a fMRI study using our paradigm in Experiment 2 in combination with the mapping technique used by Brouwer and Heeger [48] to map orientation-selective channel responses. One would expect that the response of an orientation-selective channel should be suppressed when the participant touches a grating with an orthogonal orientation. To our knowledge there are no previous accounts of crossmodal cross-orientation inhibition.

Finally, functional activation of brain areas that are usually used for visual processing has been demonstrated in blind people during Braille reading [15]–[17], [19], [20] and arises relatively quickly in normally sighted observers after several days of blindfolding [22]. Discussion of these observations generally centers on whether there are pre-existing connections from tactile to visual areas, which are usually inhibited or hidden when vision is present, or whether these cross-modal effects are due to plasticity and the growth of new connections after uni-sensory loss. Our results provide strong support for the hypothesis that there are pre-existing connections between tactile and visual areas.

## Conclusion

Our results show that congruent haptic stimulation can improve performance on a simple visual grating detection task compared to incongruent haptic stimulation. The orientation dependence of this haptic enhancement of vision suggests that neurons in the visual cortex, where orientation-tuned responses are common, receive inputs from the somatosensory cortex, likely via multisensory areas. The results cannot be due to a response bias, and are unlikely to be due to attention. Several studies suggest tactile inputs to visual cortex exist but are usually weak and masked by strong visual signals. By conducting our experiments at visual contrast threshold, the relative strength of the tactile signal has been increased to the point where it can have a small but significant enhancing effect when congruent with vision. Analogous to a visual-tactile summation model suggested by Arabzadeh and colleagues [23] for tactile tasks, we suggest that tactile signals feedback to visual cortex and sum with visual signals to increase the signal-to-noise ratio and therefore improve visual contrast sensitivity.
